# The Influence of the Type of Cement on the Properties of Surface Cement Concrete

**DOI:** 10.3390/ma15144998

**Published:** 2022-07-18

**Authors:** Tomasz Rudnicki

**Affiliations:** Faculty of Civil Engineering and Geodesy, Military University of Technology, 2 Kaliskiego St., 00-908 Warsaw, Poland; tomasz.rudnicki@wat.edu.pl

**Keywords:** concrete pavement, pore distribution in concrete, reduce the carbon footprint of concrete

## Abstract

The aim of this work was to reduce the carbon footprint of cement concrete by using multi-component cement with a high content of blast furnace slag. The analysis consisted of comparing the properties of the concrete mix and the hardened concrete made of the CEM I 42.5 R-NA cement commonly used in Poland and the CEM III cement with a large amount of blast furnace slag. The tests used cement in a constant amount of 380 kg/m^3^, granite aggregate of 2/8, 8/16, 16/22 mm and sand of 0/2 mm. As part of this project, detailed tests of the concrete mix and hardened concrete were carried out in terms of compressive, bending, fracture and frost resistance after 150 cycles of freezing and thawing, and the pore structure in hardened concrete was assessed according to PN-EN 480-11. The endurance tests were performed after 7, 28 and 90 days. On the basis of the obtained results, it was found that the highest compressive strengths above 70.2 MPa were obtained for concrete with CEM III, 64.5 MPa for concrete with CEM I. Additionally, for concrete with multi-component cement, smaller drops in compressive strength were obtained and a more favorable pore distribution in hardened concrete was obtained. Concrete intended for road surfaces can be made of both CEM I and CEM III cement, as they meet the requirements of the technical specifications for roads with heavy traffic of heavy vehicles.

## 1. Introduction

The economic development of any country depends largely on a well-developed road network. The construction of such infrastructure consumes a very large part of the expenditure of each country; therefore, it is extremely important to increase the durability of the roads built in order to reduce maintenance costs in the future and maximize the life cycle of the pavement. A reduction in maintenance costs and an extension of the life cycle of road pavements is possible thanks to the use of cement concrete pavements. In Poland, the most frequently used cement for infrastructure is Portland cement CEM I 42.5, but its production requires a lot of energy, as it contains over 90% of clinker. The activities of many researchers in the field of the properties of cement concrete mainly concerned the modification of the concrete composition through the use of various types of aggregate, the amount and type of chemical admixtures and mineral additives [1,2,3,4,5,6,7,8,9]. A reduction in CO_2_ emissions in cement concrete is most effective by using cements with the lowest possible amount of clinker [10,11,12,13,14,15,16,17]. Such a cement is CEM III, which contains 40% of clinker and 50% of blast furnace slag which is industrial waste and can significantly reduce the carbon footprint of concrete. An additional argument that strengthens the use of multi-component binders in the infrastructure of cements is a significant reduction in the possibility of an alkaline reaction in concrete. By analyzing the literature, one can see the works on the use of processed mineral waste or industrial waste [18,19,20,21,22,23,24,25,26]. A very important element of increasing the durability of concrete is to obtain the proper aeration structure through the use of air-entraining admixtures [27,28,29,30]. The tests of the concrete pavement of the A6 motorway after 80 years of operation confirmed that the tested concrete pavement is very durable, and its compressive strength parameters exceed 60 MPa [31], and the strength tests, such as frost resistance after 150 cycles and the analysis of the strength of the aeration structure meet today’s very high requirements [32,33,34,35,36]. Thanks to the optimization of the concrete mix composition in terms of the aggregates used and new solutions of chemical admixtures, we can obtain concrete surfaces with very high properties [37]. The use of admixtures reducing the amount of water and increasing the plasticity of the mix, allows for the extension of the technological process of transport and incorporation of the concrete mix by up to 2 h while maintaining the required parameters of the concrete mix during assembly [38,39,40]. The conducted tests of concrete in the field of frost resistance confirm that a well-selected air-entraining admixture is able to create an appropriate structure of air pore distribution, guaranteeing durability that exceeds the requirements for concrete surfaces [41,42,43,44]. When analyzing the life cycle of a concrete pavement, it should be emphasized that after exceeding the full service life, pavement concrete can be 100% recycled and rebuilt in the whitetopping technology [45].

## 2. Materials and Methods

### 2.1. Materials

**Figure 1:**
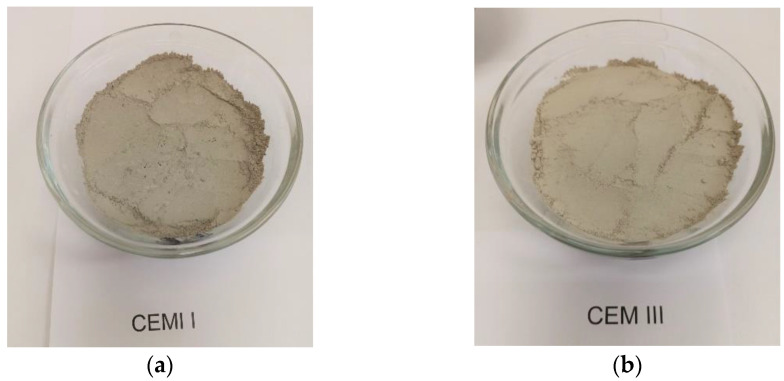
Pictures of cement samples: (**a**) CEM I 42.5 R-NA; (**b**) CEM III/A 42.5 HSR-NA.

The tests were carried out with the use of two types of cements according to PN-EN 197-1 [46], cement CEM I 42.5 R-NA and CEM III/A 42.5 HSR-NA, granite aggregate 2/8, 8/16 and 16/22, density 2.65 g/cm^3^ and water absorption 0.80% and natural sand with a fraction of 0/2 with a density of 2.64 g/cm^3^. Two types of chemical admixtures were used in the form of a high-performance/strong admixture reducing water SP of the 3rd generation, in accordance with the PN-EN 934-1 [47] and PN-EN 934-2 [48] standards, a modified high-performance polycarboxylic admixture reducing liquid water with a density of 1.02 g/cm^3^ and an aerator with a density of 1.01 g/cm^3^ and water in accordance with the requirements of PN-EN 1008 [49]. The properties and design of the cements supplied by the manufacturer are shown in Table 1 and Figure 1.

CEM I 42.5 R-NA contains more than 90% of Portland clinker (K), which is produced by sintering a precisely defined mixture of raw materials (raw meal, paste or slurry) containing elements, usually expressed as oxides, CaO, SiO_2_, Al_2_O_3_, Fe_2_O_3_ and small amounts of other materials. The raw meal, paste or slurry are finely ground, thoroughly mixed and therefore homogeneous. Portland cement clinker is a hydraulic material, at least two-thirds of its mass is composed of calcium silicates (3CaO SiO_2_ and 2CaO SiO_2_) and the remainder of aluminum and iron containing clinker phases and other compounds. The mass ratio (CaO)/(SiO_2_) must not be less than 2.0 [49]. The content of magnesium oxide (MgO) shall not exceed 5.0% (m/m). CEM III/A 42.5 HSR-NA contains more than 50% of granular blast furnace slag (S). Granular blast furnace slag is produced by rapidly cooling molten slag which has a suitable composition obtained by smelting iron ore in a blast furnace, and at least two-thirds of the mass of which is vitreous slag, and which has hydraulic properties when suitably activated. Granular blast furnace slag consists of at least two-thirds, in terms of mass, of the sum of calcium oxide (CaO), magnesium oxide (MgO) and silicon dioxide (SiO_2_). The remainder contains aluminum oxide (Al_2_O_3_) along with a small amount of other compounds. The mass ratio (CaO + MgO)/(SiO_2_) should exceed 1.0 [49].

### 2.2. Methods

The tests of the concrete mix, including the determination of air content, density, the temperature of the mix and consistency, were carried out 5 and 60 min after the completion of mixing the components in order to check the rheological behavior of the concrete mix. The vebe consistency tests were carried out in accordance with EN 12350-2 [50], the air content was determined by the pressure method in accordance with EN 12350-7 [51], and the density of the concrete mixture was determined in accordance with the EN 12350-6 [52] standard. The requirements for the concrete pavement used for the design and determination of concrete properties contained in the detailed technical specification are presented in Table 2 below:

C35/45 class concrete with a w/c coefficient of 0.40 was designed with the use of a plasticizing admixture and an air-entraining admixture. All tested concretes were made with a constant amount of cement of 380 kg/m^3^. Granite aggregate with a fraction of 2/8, 8/16 and 16/22 mm and natural sand with a fraction of 0/2 mm of the grain size are shown in Table 3.

**Figure 2:**
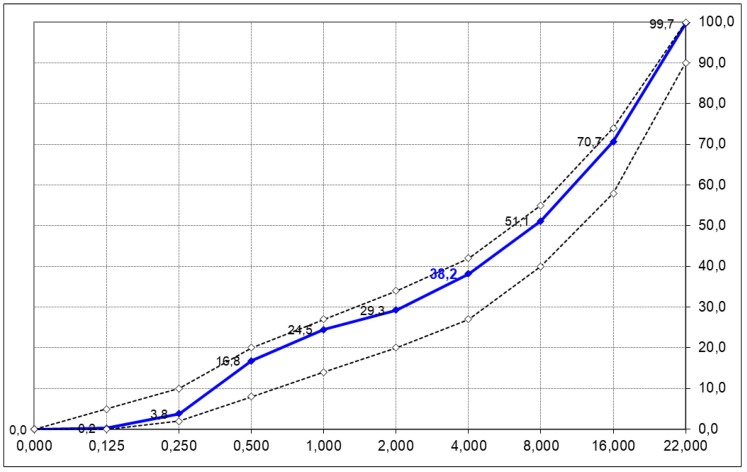
Mineral mixture grading curve.

The grading of the mineral mix along with the recommended grading curves resulting from the requirements of the technical specification are presented in Figure 2 below:

The assumptions of the research project were to determine the properties of concrete mix and hardened concrete after 7, 28 and 90 days of curing in accordance with the guidelines for using two different cements intended for use in road concrete surfaces with high traffic. The list of the compositions of the tested concrete mixtures is presented in Table 4:

The forming and storage of samples during maturation were performed in accordance with the requirements of EN 12390-2 [53]. Tests of hardened concrete in terms of determining the compressive strength, flexural strength and tensile strength were carried out in accordance with PN-EN 12390-3 [54], PN-EN 12390-6 [55], PN-EN 12390-5 [56] after 7, 28 and 90 days of maturation.

The main research element of this project was to verify the frost resistance after 150 cycles analyzed with the use of the standard method in accordance with PN-B-06250: 1988 [57] and for each series the pore distribution in hardened concrete was determined in accordance with EN 480-11 [58]. In total, more than 160 samples were prepared and tested.

## 3. Results

### 3.1. Assessment of Concrete Mix Properties

The influence of the type of cement on the properties of the concrete mix was determined 5 and 60 min after mixing the components under standard conditions. The consistency was determined in accordance with PN-EN 12350-3, the air content in the concrete mix in accordance with PN-EN 12350-7 and the density of the mix in accordance with PN-EN 12350-6. The results obtained during this study are summarized in Table 5:

Analyzing the obtained results of the consistency determination after 5 and 60 min, it can be seen that the highest parameters were obtained for the C_III concrete mix based on the CEM III cement. The concrete mix for CEM III tested after 60 min maintained the assumed consistency and air content. For the C_I mix based on CEM I, the consistency ranged from 14 s to 10 s after 60 min, and the air content from 5.8% to 5.1% after 60 min. All results were within the parameters’ range required by the specification.

### 3.2. Determination of the Compressive Strength of Concrete

**Figure 3:**
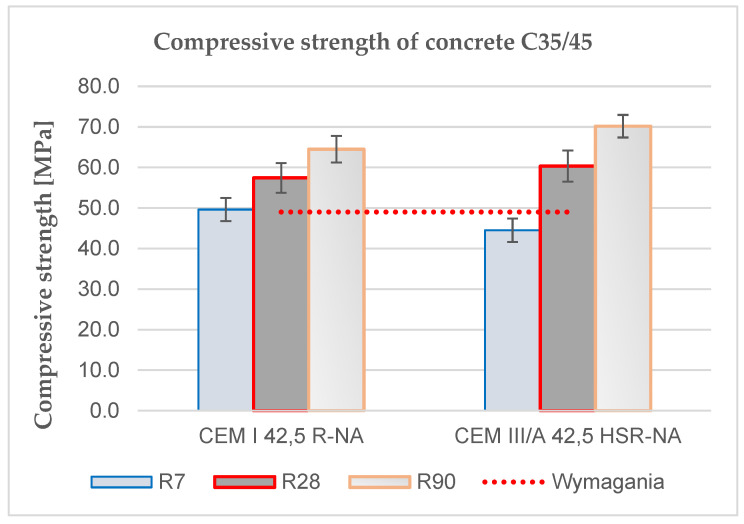
Influence of the type of cement on the determination of the compressive strength of concrete.

The effect of the type of cement on the compressive strength was determined according to PN-EN 12390-3 after 7, 28 and 90 days of maturation. The production and storage of 36 samples during the maturation period was carried out in accordance with the requirements of EN 12390-2. The average results of the obtained tests are summarized in Table 6 and Figure 3:

When analyzing the obtained results of the compressive strength test, it should be noted that the highest values were obtained for C_III concrete based on CEM III after 28 and 90 days of maturation. Concrete C_I obtained higher strength after 7 days because it is made of CEM I 42.5 R-NA cement, i.e., with early compressive strength. Regardless of the type of cement, all concretes reached the minimum of 49 MPa required by the specifications. Concrete based on CEM III cement reached 44.5, 60.3 and 70.2 MPa, respectively, after 7, 28 and 90 days of maturation. For CEM I cement, the obtained results were lower by 4.8% and 8.1% after 28 and 90 days compared to CEM III. Greater strength and greater increases in strength of concrete based on CEM III cement result from the greater amount of mineral addition in the form of blast furnace slag, which exhibits long-term binding properties. The increase in the compressive strength of the concrete with the formula C_III in the period between 28 and 90 days was 16.4%.

### 3.3. Flexural Strength of Concrete

The influence of the type of cement on the bending strength was determined according to PN-EN 12390-5 after 28 and 90 days of maturation. The production and storage of 12 samples during maturation was performed in accordance with the requirements of EN 12390-2. The average results of the obtained tests are summarized in Table 7:

The highest flexural strength results were obtained for concrete with CEM III after both 28 and 90 days of maturation. Regardless of the type of cement, all concretes reached the minimum of 5.5 MPa required by the specifications. Concrete based on CEM III cement reached 7.0 and 7.8 MPa, respectively, after 28 and 90 days of maturation. For CEM I cement, the obtained results were lower by 2.9% and 10.3% compared to CEM III. The increase in the strength of concrete with formula C_III in the period from 28 to 90 days was 11.4%.

### 3.4. Determination of Concrete Tensile Splitting Strength of the Test Specimens 

The determination of the impact of the type of cement on the tensile splitting strength of the test specimens was performed in accordance with PN-EN 12390-6 after 28 and 90 days of maturation. The production and storage of 12 samples during maturation was compliant with the requirements of EN 12390-2. The average results of the obtained tests are summarized in Table 8:

The highest tensile splitting strength of the test specimens was achieved for C_I concrete after both 28 and 90 days. Regardless of the type of cement, all concretes reached the minimum of 3.5 MPa required by the specifications.

### 3.5. Determination of Frost Resistance after 150 Cycles

Concrete frost resistance was tested in accordance with PN-B-06250:1988 using the standard method, the procedure for F150 freezing and thawing cycles. From each of the tested mixtures, 12 cubes with the side length of 100 mm were formed. The produced specimens were then immersed in water for 28 days. Six samples of each blend were then weighed and subjected to 150 freeze–thaw cycles. The remaining six samples were left immersed in water as reference samples. After 150 freeze–thaw cycles, the samples were weighed and visually inspected. The compressive strength of all samples was determined as the last step in the test procedure. According to the frost resistance criteria, PN-B-06250: 1988, the weight variation cannot exceed 5%, and the loss of compressive strength cannot exceed 20% in reference to samples made of the same mixture that have not been subjected to cyclic freezing and thawing. Moreover, the samples after testing must not show any cracks. The test results are summarized in Table 9 below:

When analyzing the obtained results of strength tests in the form of frost resistance after 150 cycles, it should be noted that the smallest decrease in compressive strength after 150 cycles of freezing and thawing, amounting to 4.1%, was obtained for concrete C3_III. The tested pavement concretes based on CEM I and CEM III met the condition required in the technical specification because the decrease in compressive strength was less than 20% and the weight loss of the samples was less than 5%.

### 3.6. Air Void Analysis

**Figure 4:**
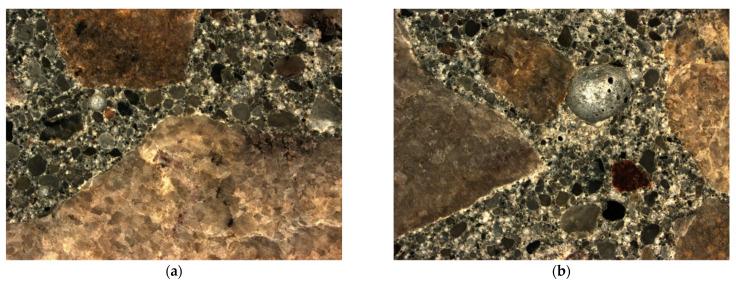

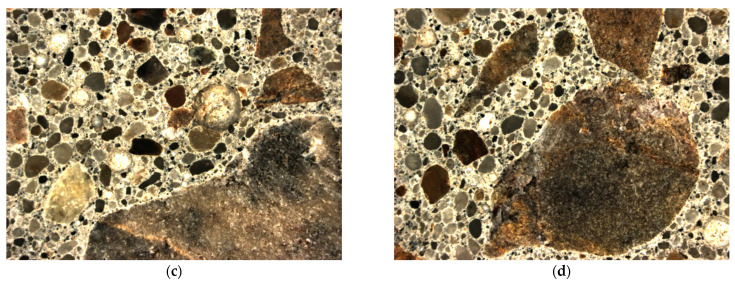
The image of the tested concrete samples after cutting: (**a**) and (**b**) C_I; (**c**) and (**d**) C_III.

The characteristics of air voids in hardened concrete were determined in accordance with EN 480-11: 2008. Test specimens with dimensions of 150 × 100 × 20 mm were cut from cubes with a side length of 150 mm formed in the laboratory. The void characteristics were measured by the microscopic method using a computer system for automatic image analysis, Nikon SMZ1270 Navitar. Two samples of each tested concrete were selected for testing the properties of air voids. The image of the hardened concrete structure is shown in Figure 4.

To determine the spacing of air voids, a calculation model was adopted, which assumes the presence of air voids of a specific diameter. It is a transitional model between the real state and the Powers model. The results of the air voids distribution in the hardened concrete are presented in Table 10.

The obtained average values of the analyzed parameters, determined in accordance with the EN 480-11: 2008 [58] standard in individual tests, are presented in Table 10 Each result is the average of 4 tested samples. By analyzing the obtained results of determining the characteristics of the spacing of air voids, it can be concluded that they meet the requirements for pavement concrete used in XF conditions.

The total air content is similar to the results obtained in the concrete mix air content method, determined by the pressure method (from 5.1% to 5.8%). The total air content in the hardened concrete ranges from 4.71% to 5.77%. The highest value of the air gap coefficient (*L*) and the lowest micro air-void content (A_300_) were demonstrated by the concrete marked C_III based on the CEM III cement.

## 4. Discussion

When analyzing the obtained test results, it should be stated that the pavement concrete with the use of CEM III/A 42.5 HSR-NA cement meets very high requirements for a modern concrete pavement intended for heavy traffic on motorways. The aim of the research work was to check the properties of concrete for two different cements, CEM I 42.5 R-NA, commonly used and required in Poland, and CEM III/A 42.5 HSR-NA, which is a very interesting alternative, especially in terms of reducing the carbon footprint and environmental protection. The composition of CEM III cement contains over 45% of blast furnace slag which is a waste material, and thanks to this we can reduce the carbon footprint and CO_2_ emissions by over 30%. By analyzing the individual properties of the concrete mix, it can be stated that the C_III concrete mix has better rheological properties after both 5 and 60 min from mixing the components. Analyzing the results of strength tests, it can be concluded that the concrete with CEM III has a compressive strength higher by 9% after 90 days of maturation. The use of CEM III cement for the construction of cement concrete road pavements is a beneficial alternative for the future, as it results in the reduction in the carbon footprint and lower CO_2_ emissions [10], at the same time allowing for the use of waste material such as blast furnace slag without losing the properties of concrete. The durability of pavement concrete verified during F150 frost resistance tests and the analysis of the air-entrainment structure clearly indicate the possibility of using CEM III/A 42.5 HSR-NA cement for the construction of concrete pavements.

## 5. Conclusions

Based on the tests carried out for the C35/45 class pavement concrete tested on two different cements, CEM I 42.5 R-NA and CEM III/A 42.5 HSR-NA, the following conclusions can be drawn:

1. For both cements, the specification requirements were met, both in terms of strength tests and durability tests.
2. The use of CEM III multi-component cement in concrete allowed to achieve 71 MPa of compressive strength after 90 days of maturation, i.e., 9% more than for CEM I.
3. The use of CEM III cement improved frost resistance, as the decrease in compressive strength was 4.1%, and 10.5% for concrete with CEM I.
4. Concrete with CEM III multi-component cement achieved a better pore structure in the form of A300—3.15% and L—0.07.
5. Multi-component cement increases the filling of the cement paste structure, and thus increases the durability of cement concrete by reducing the loss of strength by up to 60%.

## Figures and Tables

**Table 1:** Properties of cements provided by the manufacturer.

Property	Unit	Type of Cement	
		CEM I 42.5 R-NA	CEM III/A 42.5 HSR-NA
**Water Lust**	%	25.8	29.8
Seting time initial	min	205	238
Seting time final	min	274	317
Consistency in volume	mm	0.8	0.8
Specific surface	cm^2^/g	3275	4466
Compressive strength F_2_	MPa	26.8	14.2
Compressive strength F_28_	MPa	54.7	51.5
LOI	%	3.37	2.61
IR	%	0.66	0.82
SiO_2_	%	18.95	29.18
Al_2_O_3_	%	4.99	6.17
Fe_2_O_3_	%	2.81	1.55
CaO	%	62.82	50.54
MgO	%	1.37	4.04
SO_3_	%	3.14	2.48
Na_2_O	%	0.21	0.33
K_2_O	%	0.88	0.69
eqNa_2_O	%	0.59	0.78
Cl^−^	%	0.08	0.07

**Table 2:** Requirements for the concrete pavement for traffic categories KR5—KR7.

Properties of Concrete Pavement	Requirements	Test Method
Density, tolerance in reference to the formula	±3.0%	PN-EN 12390-7
Compressive strength class for traffic category KR5—KR7, not lower than:	C35/45	PN-EN 12390-3
Flexural strength of concrete for traffic category KR5—KR7, not lower than:	5.5 MPa	PN-EN 12390-5
Tensile strength of concrete when splitting for traffic category KR5—KR7, not lower than:	3.5 MPa	PN-EN 12390-6
Characteristics of air pores in concrete:		PN-EN 480-11
− the content of micropores with a diameter below 0.3 mm (A300), % − index of the distribution of pores in concrete, *L* mm	≥1.5% ≤0.200 mm	
Concrete frost resistance test F150:		PN-B-06250
− weight loss of the sample, not more than, % − decrease in compressive strength, no more than, %	5 % 20%	

**Table 3:** Graining of materials.

Sieve	Screening [%]			
[mm]	Granite 16/22	Granite 8/16	Granite 2/8	Sand 0/2
22.000	1.0	0.0	0.0	0.0
16.000	95.5	1.7	0.00	0.0
8.000	3.5	96.3	1.2	0.0
4.000	0.0	2.0	59.70	0.0
2.000	0.0	0.0	39.10	2.5
1.000	0.0	0.0	0.0	15.9
0.500	0.0	0.0	0.0	25.5
0.250	0.0	0.0	0.00	43.4
0.125	0.0	0.0	0.0	11.9
0.000	0.0	0.0	0.0	0.8
**sum:**	**100.0**	**100.0**	**100.0**	**100.0**

**Table 4:** Composition of concrete mixtures.

Materials	Concrete Mix Compositions [kg/m^3^]	
	C_I	C_III
CEM I 42.5 R	**380**	-
CEM III A 42.5 HSR-NA	-	**380**
Water	152	152
Sand 0/2	573	573
Granite 2/8	401	401
Granite 8/16	363	363
Granite 16/22	573	573
SP PC	3.04	3.04
LPA	0.76	0.76
Density	2446.1	2446.1

**Table 5:** Properties of the concrete mix.

Materials	Composition of Concrete Mixtures [kg/m^3^]	
	C_I	C_III
Consistency after 5 min, s	14	15
Consistency after 60 min, s	10	14
Air content after 5 min, %	5.8	5.6
Air content after 60 min, %	5.1	5.4
Density, g/cm^3^	2.440	2.442

**Table 6:** Influence of the type of cement on the compressive strength after 7, 28 and 90 days of maturation.

Materials	Compressive Strength [MPa]	
	C_I	C_III
Compressive strength after 7 days	49.6	44.5
Compressive strength after 28 days	57.4	60.3
Compressive strength after 90 days	64.5	70.2
Density, g/cm^3^	2.442	2.447

**Table 7:** Influence of the cement type on flexural strength after 7, 28 and 90 days of maturation.

Materials	Flexural Strength [MPa]	
	C_I	C_III
Flexural strength after 28 days	6.8	7.0
Flexural strength after 90 days	7.0	7.8

**Table 8:** Influence of the type of cement on the tensile splitting strength of the test specimens after 28 and 90 days.

Materials	Tensile Splitting Strength of the Test Specimens [MPa]	
	C_I	C_III
Concrete tensile splitting strength of the test specimens after 28 days	4.5	4.4
Concrete tensile splitting strength of the test specimens after 90 days	5.4	5.2

**Table 9:** Influence of the cement type on frost resistance after 28 days of maturation.

Frost Resistance Test F150	Type of Mixture	
	C_I	C_III
Mean decrease in the strength of specimens ΔR, %	10.5	4.1
Mass change of specimens subjected to cyclical freezing and thawing ΔG, %	0.03	0.02

**Table 10:** The average values of the parameters characterizing air voids and their spacing.

Parameter	Unit	C_I	C_III
Total air content, *A*	%	4.71	5.77
Spacing factor, *L*	mm	0.16	0.07
Micro air-void content, *A*_300_	%	2.49	3.15
Specific surface of the air	mm^−1^	48.6	56.8
Total traverse length	mm	2646	2646
Total number of chords measured		3214	3214

## Data Availability

Not applicable.

## References

[1] Glinicki M.A. (2019). Inżynieria Betonowych Nawierzchni Drogowych.

[2] Glinicki M.A. (2019). Właściwości betonu nawierzchniowego z kruszywem odkrytym—Wpływ rodzaju cementu i pielęgnacji. Drog. LXXIV.

[3] Hu M.S., Siddiqui D.W., Fowler D. Whitney, Two-Lift Concrete Paving—Case Studies and Reviews from Sustainability, Cost Effectiveness and Construction Perspectives. Proceedings of the TRB 2014 Annual Meeting.

[4] Pranav S., Aggarwal S., Yang E.H., Sarkar A.K., Singh A.P., Lahoti M. (2020). Alternative materials for wearing course of concrete pavements: A critical review. Constr. Build. Mater..

[5] Rudnicki T. (2016). The method of aggregate skeleton in self compacting concrete designing with segment regression. Cem. Wapno Beton.

[6] Mackiewicz P., Szydło A., Krawczyk B. (2018). Influence of the construction technology on the texture and roughness of concrete pavements. Roads Bridges Drog. Mosty.

[7] Jurczak R., Szmatuła F., Rudnicki T., Korentz J. (2021). Effect of Ground Waste Glass Addition on the Strength and Durability of Low Strength Concrete Mixes. Materials.

[8] Glinicki M.A., Dąbrowski M., Skrzypczyński M. (2017). Influence of curing on the properties of air-entrained concrete in the upper layer of exposed aggregate pavement-modelling study. Cem. Wapno Beton.

[9] Małek M., Jackowski M., Łasica W., Kadela M. (2021). Influence of Polypropylene, Glass and Steel Fiber on the Thermal Properties of Concrete. Materials.

[10] Institute of Building Technique, Department of Heat Physics, Sanitary Installations and Environment Pracownia Ochrony Środowiska: Performing a life cycle analysis (LCA) to determine the carbon footprint for medium cements from the CEM I, CEM II and CEM III groups produced in Poland, in accordance with PN-EN 15804: 2012 work number: 01929/12/ZOONF.

[11] Kaszuba S. (2019). Kształtowanie Składu Trwałego Betonu z Udziałem Cementów Wieloskładnikowych (CEM II, CEM III) do Zastosowania w Budownictwie Drogowo-Mostowym.

[12] Dam V., Taylor P., Fick G., Gress D., van Geem M., Lorenz E. (2011). Sustainable Concrete Pavements: A Manual of Practice.

[13] Giergiczny Z., Glinicki M.A., Sokołowski M., Zieliński M. (2009). Air void system and frost salt scaling of concrete containing slag blended cement. Constr. Build. Mater..

[14] Shubbar A.A., Jafer H., Dulaimi A., Hashim K., Atherton W., Sadique M. (2018). The development of a low carbon binder produced from the ternary blending of cement, ground granulated blast furnace slag and high calcium fly ash: An experimental and statistical approach. Constr. Build. Mater..

[15] Yildirim H., Ilica T., Sengul O. (2011). Effect of cement type on the resistance of concrete against chloride penetration. Constr. Build. Mater..

[16] Rahhal V., Bonavetti V., Trusilewicz L., Pedrajas C., Talero L. (2012). Role of the filler on Portland cement hydration at early ages. Constr. Build. Mater..

[17] Ghrici M., Kenai S., Said-Mansour M. (2007). Mechanical properties and durability of mortar and concrete containing natural pozzolana and limestone blended cements. Cem. Concr. Compos..

[18] TL Beton-StB (2007). Technische Lieferbedingungen für Baustoffe und Baustoffgemische für Tragschichten mit hydraulischen Bindemitteln und Fahrbahndeckenaus Beton.

[19] Breitenbücher R., Costner C. (2013). Waschbetonoberflächen, Mindestluftporengehalt in Waschbeton. Forschung Straßenbau und Straßenverkhertechnik.

[20] Rudy A., Olek J., Nantung T., Newell R.M., Brandt A.M., Olek J., Marshall I.H. (2009). Statistical Optimization of Low Slump Ternary Concrete Mixtures with Ground Granulated Blast Furnace Slag (GGBS) and High Calcium Fly Ash for Pavement Applications, Brittle Matrix Composites-9.

[21] Babu K.G., Kumar V.S.R. (2000). Efficiency of GGBS in concrete. Cem. Concr. Res..

[22] Rudnicki T. (2021). Functional Method of Designing Self-Compacting Concrete. Materials.

[23] Małek M., Łasica Ł., Kadela M., Kluczyński J., Dudek D. (2021). Physical and Mechanical Properties of Polypropylene Fibre-Reinforced Cement–Glass Composite. Materials.

[24] Horszczaruk E., Brzozowski P., Adamczewski G., Rudnicki T. (2014). Influence of Hydrostatic Pressure on Compressive Strength of Self-Consolidating Concrete. J. Civ. Eng. Archit..

[25] Rudnicki T., Jurczak R. (2022). The impact of the addition of diabase dusts on the properties of cement pavement concrete. Arch. Civ. Eng..

[26] Małek M., Kadela M., Terpiłowski M., Szewczyk T., Łasica W., Muzolf P. (2021). Effect of metal lathe waste addition on the mechanical and thermal properties of concrete. Materials.

[27] Łaźniewska-Piekarczyk B., Gołaszewski J. (2019). Relationship Between Air-Content in Fresh Cement Paste, Mortar, Mix and Hardened Concrete Acc. to PN-EN 480-1 with Air-Entraining CEM II/BV. IOP Conf. Ser. Mater. Sci. Eng..

[28] Tunstall L.E., Ley M.T., Scherer G.W. (2021). Air entraining admixtures: Mechanisms, evaluations, and interactions. Cem. Concr. Res..

[29] Dziedzic K., Dąbrowski M., Antolik A., Glinicki M.A. (2020). Characteristics of concrete mix air-entrainment applying the sequential pressure method. Roads Bridges Drog. I Mosty.

[30] Wong H.S., Pappas A.M., Zimmerman R.W., Buenfeld N.R. (2011). Effect of entrained air voids on the microstructure and mass transport properties of concrete. Cem. Concr. Res..

[31] Rudnicki T., Jurczak R. (2020). Recycling of a Concrete Pavement after over 80 Years in Service. Materials.

[32] Szydło A., Mackiewicz P., Wardęga R., Krawczyk B. (2014). Katalog typowych konstrukcji nawierzchni sztywnych. Załącznik do zarządzenia Nr 30 Generalnego Dyrektora Dróg Krajowych i Autostrad. Warszawa.

[33] Glinicki M.A., Jaskulski R., Dąbrowski M. (2016). Design principles and testing of internal frost resistance of concrete for road structures-critical review. Roads Bridges Drog. I Mosty.

[34] Liu Z. (2014). Frost Deterioration in Concrete Due to Deicing Salt Exposure: Mechanism, Mitigation and Conceptual Surface Scaling Model. Ph.D. Thesis.

[35] GDDKiA (2019). Warunki Wykonania i Odbioru Robot Budowlanych D-05.03.04 v2 Nawierzchnia z Betonu Cementowego.

[36] (2014). Catalogue of Typical Structures of Rigid Pavements. GDDKiA, Warszawa, GDDKiA. https://www.gddkia.gov.pl/frontend/web/userfiles/articles/d/dokumenty-techniczne_8162/Dokumenty%20techniczne/KTKNS.pdf.

[37] Jasiczak J., Wdowska A., Rudnicki T. (2008). Ultra-High Performance Concretes. Properties, Technology, Applications.

[38] Gołaszewski J. (2016). Domieszki do betonu. Efekt działania, ocena i badania efektywności, stosowanie.

[39] Rudnicki T. (2004). Natural and synthetic admixtures plasticize the tail and mechanisms of their interaction in the concrete mix. Mag. Autostrady.

[40] Pasławski J., Rudnicki T. (2021). Agile/Flexible and Lean Management in Ready-Mix concrete delivery. Arch. Civ. Eng..

[41] Breitenbücher R., Bou-Young Y. (2010). Qualitätssicherung von Waschbetonoberflächen, Berichte der Bundesanstalt für Straßenwesen. Straßenbau Heft S.

[42] Giergiczny Z., Baran T., Dziuk D., Ostrowsk M. (2016). The increase of concrete frost resistance by using cement with air-entraining agent. Cem. Wapno Beton.

[43] Glinicki M.A. (2014). Methods of qualitative and quantitative assessment of concrete air entrainment. Cem. Wapno Beton.

[44] Dziedzic K., Dąbrowski M., Antolik A., Glinicki A. (2020). Charakterystyka napowietrzenia mieszanki betonowej metodą sekwencyjno-ciśnieniową. Roads Bridges Drog. I Mosty.

[45] Rudnicki T., Wołoszka P. (2016). The use of technology whitetopping in the aspect of implementation of repairs of flexible pavements. Bull. Mil. Univ. Technol..

[46] Cement—Część 1: Skład, Wymagania i Kryteria Zgodności Dotyczące Cementów Powszechnego Użytku.

[47] Domieszki do Betonu, Zaprawy i Zaczynu—Część 1: Wymagania Podstawowe.

[48] Domieszki do Betonu, Zaprawy i Zaczynu—Część 2: Domieszki do Betonu. Definicje, Wymagania, Zgodność, Znakowanie i Etykietowanie.

[49] Woda Zarobowa do Betonu.

[50] (2019). Testing Fresh Concrete—Part 2: VeBe consistency testing.

[51] (2019). Testing Fresh Concrete. Air content. Pressure Methods.

[52] (2019). Testing Fresh Concrete. Density.

[53] (2019). Testing Hardened Concrete—Part 2: Making and Curing Specimens for Strength Tests.

[54] (2019). Testing Hardened Concrete—Part 3: Compressive Strength of Test Specimens.

[55] (2019). Testing Hardened Concrete—Part 6: Bending Strength of Test Specimens.

[56] (2019). Testing Hardened Concrete—Part 5: Tensile Strength when Splitting Test Specimens.

[57] (1988). Polish Standard Normal Concrete (in Polish).

[58] (2008). Admixtures for Concrete, Mortar and Grout-Test Methods—Part 11: Determination of Air Void Characteristics in Hardened Concrete.

